# Cochlear Outer-Hair-Cell Power Generation and Viscous Fluid Loss

**DOI:** 10.1038/srep19475

**Published:** 2016-01-21

**Authors:** Yanli Wang, Charles R. Steele, Sunil Puria

**Affiliations:** 1 Mechanical Engineering, Stanford University, Stanford, CA, USA; 2 Otolaryngology–Head and Neck Surgery, Stanford University, Stanford, CA, USA

## Abstract

Since the discovery of otoacoustic emissions and outer hair cell (OHC) motility, the fundamental question of whether the cochlea produces mechanical power remains controversial. In the present work, direct calculations are performed on power loss due to fluid viscosity and power generated by the OHCs. A three-dimensional box model of the mouse cochlea is used with a feed-forward/feed-backward approximation representing the organ of Corti cytoarchitecture. The model is fit to *in vivo* basilar membrane motion with one free parameter for the OHCs. The calculations predict that the total power output from the three rows of OHCs can be over three orders of magnitude greater than the acoustic input power at 10 dB sound pressure level (SPL). While previous work shows that the power gain, or the negative damping, diminishes with intensity, we show explicitly based on our model that OHC power output increases and saturates with SPL. The total OHC power output is about 2 pW at 80 dB SPL, with a maximum of about 10 fW per OHC.

The cochlea is an intricate biomechanical apparatus that converts the mechanical energy of vibration into electrochemical energy to stimulate auditory nerve fibers. The ability of mammals to hear high-frequency sounds is attributed to a sophisticated mechanical Fourier-transform-like decomposition of sound into a frequency-dependent traveling wave formed by the interation of cochlear fluid and the basilar membrane (BM)^1^. Upon stimulation of the organ of Corti (OoC), which is attached to the top of the BM, the outer hair cells (OHCs) exhibit piezoelectric behavior that amplifies and sharpens the traveling wave to provide the high sensitivity and sharp frequency selectivity of the mammalian cochlea. The OHCs underlie cochlear compressive nonlinearity with respect to the input sound pressure level (SPL), which allows an increase in sensitivity at lower SPLs without causing damage at higher SPLs, i.e., it provides disproportionately higher magnitude gain at lower SPLs and almost no gain at high SPLs.

Ever since the discovery of the active mechanisms in living cochleae^2^ and the motility of the OHCs^3^,^4^,^5^, there has been heated discussion as to whether or not the cochlea provides mechanical power^6^,^7^,^8^,^9^. De Boer and Nuttall^7^ and Lukashkin *et al*.^8^ conclude that there is power amplification of the traveling wave, while van der Heijden and Versteegh^9^ recently claim evidence against this. Despite decades of intense effort, direct experimental support for cochlear power gain is still lacking due to the difficulties in measuring energy flow, since both velocity and pressure measurements are required. Approximations of the pressure by measuring the BM velocity and using an assumed local BM impedance have been attempted^10^, however such formulations and calculations have been shown to be misleading^11^. An innovative method has been to approximate the BM displacement by calculating the spatial pressure gradient at multiple locations^12^,^13^. Experiments have also been performed to measure the BM displacement and pressure in the SV simultaneously^14^. However, these experimental results are inconclusive for power gain.

De Boer and Nuttall^7^ were, to our knowledge, the first to compute the power flux (the power acting on a cross section of the cochlear duct) along the length of the cochlea for passive and active cochleae at low SPLs, using an inverse method. Changes in the power flux are only the net effect of OHC power input and energy dissipation within the fluid. Comparisons of the net power change between active and passive cases, and between different SPLs, are not accurate indicators of OHC power input without also knowing the energy dissipated. For example, even though an increase in power on a cross section indicates an injection of power^7^, this power increase is not equal to the power output from the OHCs. More importantly, a decrease in power on a cross section, which is the case for both passive and active cases at high SPLs, does not mean that the power input from the OHCs is zero in both cases.

The current work is, as far as we know, the first to separate the OHC power input and energy dissipation from the net change of power on a cross section, and is completed by a forward calculation based on a 3D model of the cochlea with physiological variations in geometry, fluid viscosity, and consideration made to the cytoarchitecture of the organ of Corti. The calculation of power and energy employs numerical integration that accounts for the non-uniform 3D distribution of fluid velocity and pressure in the cochlear ducts. In agreement with previous work^7^,^14^,^15^, we show a positive net power gain basal to the best-frequency (BF) location at lower SPLs, and the maximum power on a cross section at the BF location. From calculations based on our model, at a low input level such as 10 dB SPL, the mechanical power provided by the three rows of OHCs is more than three orders of magnitude larger than the input acoustic power at the stapes. Our quantitative study of cochlear power flow was also performed for a range of higher stimulus intensities. Previous study on the effects of input intensity have reached the conclusion that, with increasing SPL the amplifier becomes less active and has a smaller effect on BM impedance in comparison to the low SPL case^16^. We show quantitatively based on our model, for the first time, that as the input SPL, increases, the absolute power provided by the OHCs continues to grow and eventually saturates, even though the relative power output of the OHCs decreases with respect to the acoustic input. The model predicts that the maximum power output provided per OHC is about 10 fW (1 fW = 10^−15^ W) at 80 dB SPL.

## Results

### Cochlear model and data fitting

**Figure 1:**
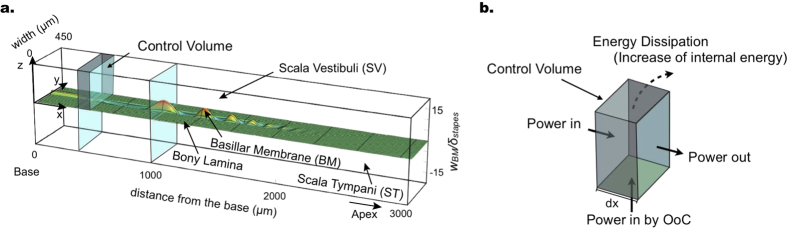
Demonstration of an instantaneous waveform on the basilar membrane (BM) in the box model (left), and energy balance for a control volume (right). (**a**) The normalized instantaneous waveform on the BM in the box model is in response to a 35 kHz input stimulus in a passive cochlea. The vertical scale on the right side of the box indicates the BM displacement (*w*_BM_) normalized by the magnitude of the stapes displacement (δ_stapes_). The power on each cross section along the cochlea is calculated as a function of *x*. Two cross sections are shown for demonstration purposes. Note that only the basal half of the total length (6.8 mm) of the mouse cochlea is shown, since the wave amplitude becomes negligible in the apical half for this frequency. (**b**) The example control volume in (**a**) is enlarged to show how the conservation of energy is analyzed.

The cochlea is modeled as a rectangular box with rigid walls. The volume of the box model is divided longitudinally into an upper duct and a lower duct, representing the scala vestibuli (SV) and scala tympani (ST) respectively, and the mechanical effect of the Reissner’s membrane is neglected. The partition between the SV and ST consists of rigid laminae along its sides and a flexible plate with changing width in between to represent the BM, as shown in Fig. 1a. The anatomical parameters were chosen to represent the mouse cochlea (see Table 1 in Methods)^17^. The two ducts are filled with fluid matching the density and viscosity of water at the mouse body temperature^18^. The organ of Corti is modeled using the feed-forward/feed-backward (FF/FB) approximation for the activity of the OHCs within the OoC cytoarchitecture^19^,^20^, while the vibration of the BM and fluid are solved using the WKB solution method in the frequency domain^21^. Details of the solution method for a box model with changing geometry, viscous fluid, and a FF/FB mechanism can be found in our previous work^19^,^20^,^21^. An improvement made in the current work is to include an additional no-slip boundary condition on the top wall of the SV. The energy in the cochlea and the power output of the OHCs are then analyzed using conservation of energy. The calculations of power on a cross section and energy dissipation are performed using numerical integration with 3D distributions of velocity and pressure within the fluid (see Methods).

**Figure 2:**
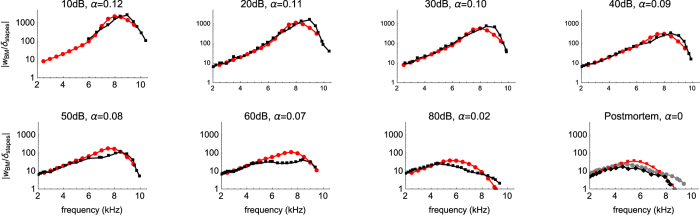
Basilar membrane (BM) displacement (*w*_*BM*_) normalized by stapes displacement (δ_sta**pes**_). The red connected markers are modeling results and black connected markers are the experimental data from Lee *et al*. 2015^25^, measured *in vivo* on intact adult mouse cochleae using optical coherence tomography. Different *α* values are mapped to different SPLs by comparing the modeling results to the experimental measurements.

In this work a single free parameter is determined by data fitting, the non-dimensional OHC force-conversion factor *α*, which is the ratio of the OHC output axial force to the input shear force at the OHC hair bundle^22^. Combined with an approximation of the OoC cytoarchitecture^23^,^24^, the force-conversion factor *α* provides the amplification of the BM displacement in the model. A comparison between the modeling results and the measurements of the normalized BM displacement allows the *α* values to be mapped to different SPLs, as shown in Fig. 2 for seven values from 10–80 dB SPL as well as for the postmortem condition. The experimental data were obtained *in vivo* using optical coherence tomography (OCT) on an intact adult mouse cochlea over the 2–10 kHz frequency region^25^. The modeling calculations correspond to a location 5.6 mm from the stapes, which best fits the measured data. The calculation of power is done for 10 kHz and 35 kHz, the latter of which lies in the middle of the mouse hearing range. The *α–*SPL mapping for 35 kHz is estimated using this mapping for the 2–10 kHz range, under the assumption that a similar peak BM displacement would be achieved at 35 kHz^26^.

### Pressure, velocity, and power distributions

**Figure 3:**
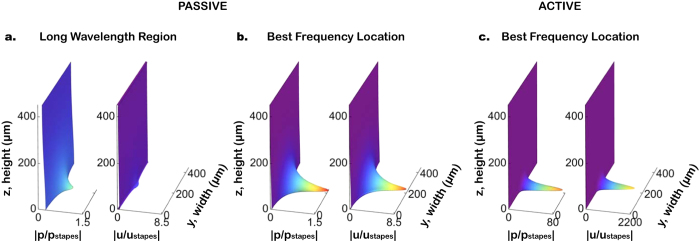
Distributions of fluid pressure and fluid velocity used for accurate calculation of power. (**a**) The normalized pressure (left) and normalized velocity (right) for a passive cochlea in the long-wavelength region. (**b**) Corresponding results for a passive cochlea at the best-frequency (BF) location. (**c**) Results for an active cochlea at the BF location with *α* = 0.08 (corresponding to a 20 dB SPL input; note the different scales). The *y* and *z* axes are the width and height respectively of the scala vestibuli (SV) as in Fig. 1, with the BM located at *z* = 0. The responses are all for a 35 kHz input stimulus. The rainbow color-coding and horizontal scales indicate the magnitude of the pressure or velocity as normalized by the corresponding value at the stapes.

Spatial distributions of pressure *p* and fluid velocity *u*, in the *x* direction, on cross sections of the SV for a 35 kHz input, are shown in Fig. 3 for two different locations along the BM length: at the BF location (Fig. 3b,c), where the wavelength is relatively short for both passive and active cases; and (for the passive case) at a basal location a quarter of the distance from the stapes to the BF location (Fig. 3a), where the wavelength is longer. With *α* = 0.08, corresponding to a sound stimulus of 10 dB SPL, the pressure and velocity are magnified significantly, as can be seen by comparing Fig. 3b,c (note the different plotting scales). It can also be seen that the pressure and velocity distributions are both concentrated near the BM, which is especially prominent at the BF location (Fig. 3b,c) as compared to the long-wavelength region (Fig. 3a). Despite large velocity amplitudes in the longitudinal and radial directions near the BM, the velocities in these directions rapidly go back to zero at the bottom and the ceiling of the SV due to the no-slip boundary conditions enforced by equation (1) in the Methods. The concentrated distributions of the fluid pressure and fluid velocity in Fig. 3 illustrate the 3D nature of the cochlear response that needs to be considered for accurate power calculations.

**Figure 4:**
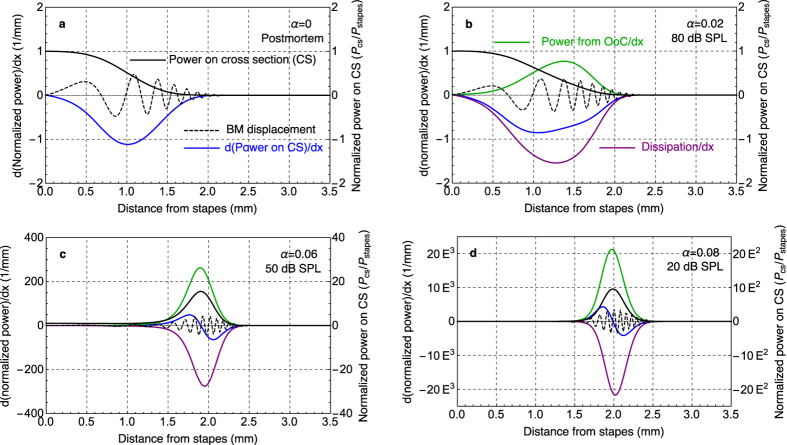
Normalized power distributions along the length of the cochlea for four amplification levels, at 35 kHz. Each subplot shows (1) the non-dimensional time-averaged power acting on SV cross sections (solid black lines); (2) the derivative of the power with respect to distance *x* from the stapes, giving the power change per unit length along the cochlea (blue lines), which is also the net effect of (3) the power loss per unit length due to the viscosity of the fluid (purple lines) and (4) the power output of the OoC per unit length (green lines); and (5) a snapshot of the BM waveform, which is rescaled arbitrarily for visibility, is shown on the same plot (dashed black lines). The scale of (1), the normalized power on each cross section (CS), is shown on the right of each plot, and the scale of (2), (3), and (4), representing the change in the normalized power per unit length, is shown on the left of each plot with units of 1/mm. Panel (**a**) contains responses for a passive postmortem cochlea (*α* = 0), (**b**) for a low level of force conversion (*α* = 0.02, 80 dB SPL), (**c**) for a medium level of force conversion (*α* = 0.06, 50 dB SPL), and (**d**) for a high level of force conversion (*α* = 0.08, 20 dB SPL). Note that only the basal half of the mouse cochlea is shown.

With the 3D distribution of the fluid pressure and fluid velocity known, the power on each cross section along the cochlea was calculated for four different force-conversion factors, corresponding to four different SPLs, using a single input tone of 35 kHz. Fig. 4 shows (1) the normalized time-averaged power on cross sections (solid black lines); (2) the change of power per unit length, i.e., the spatial derivative of (1) (blue lines = d (solid black lines)/dx), which is, from conservation of energy, the sum of (3) the power loss per unit length due to fluid viscosity (purple lines) and (4) the OHC power output per unit length (green lines); and (5), for reference, the instantaneous waveform of the BM displacement, *w*_BM_, rescaled differently for visibility (dashed black lines). The power on the cross sections is normalized with respect to the power input at the stapes at each input level. For the passive postmortem case in Fig. 4a, the normalized power on the cross sections (solid black) monotonically decreases from 1 at the stapes to 0 with increasing distance from the base. This is expected because for a passive system (*α* = 0) there is no OHC power output, and only viscous loss (coinciding with the blue line for this case) contributes to the change of power per unit length (blue). For the active cases (*α* > 0), the normalized power on the cross sections (solid black) either decreases more gradually from base to apex when the input level is high and force-conversion factor is low (Fig. 4b with *α* = 0.02), or increases and then decreases when the input level is low and force-conversion factor is higher (Fig. 4c,d with *α* = 0.06 and 0.08 respectively). It can be seen that for *α* = 0.08 (Fig. 4d), the normalized power loss per unit length (purple) and the normalized OHC power output per unit length (green) are significant and comparable to each other. The sum of the two (blue = purple + green) is relatively small, but nevertheless shows a positive region (between approximately 1.6 and 2 mm in Fig. 4d), indicating net power gain basal to the BF location. This region is often referred to as the region of negative damping^7^,^14^,^15^. Moreover, for the active cases, the viscous loss (purple) is considerably higher than the passive postmortem case (coinciding with the blue line in Fig. 4a) due to greater motion of the fluid and the BM.

### Power output of a single outer hair cell

**Figure 5:**
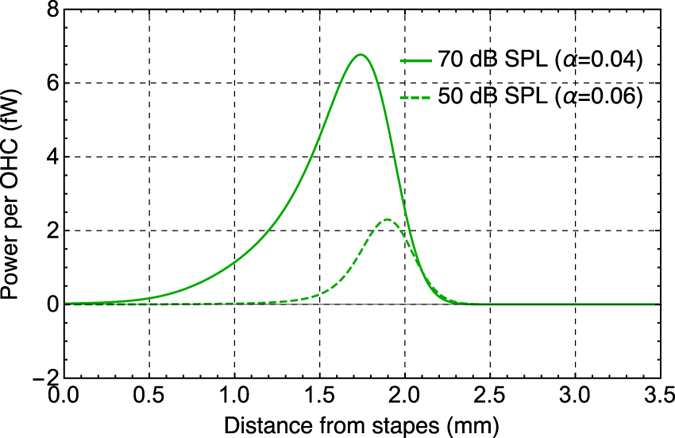
Power-output per OHC along the length of the cochlea (in fW) for two different OHC force-conversion factors *α*, with a 35 kHz stimulation. Note that only the basal half of the cochlea is shown.

**Figure 6:**
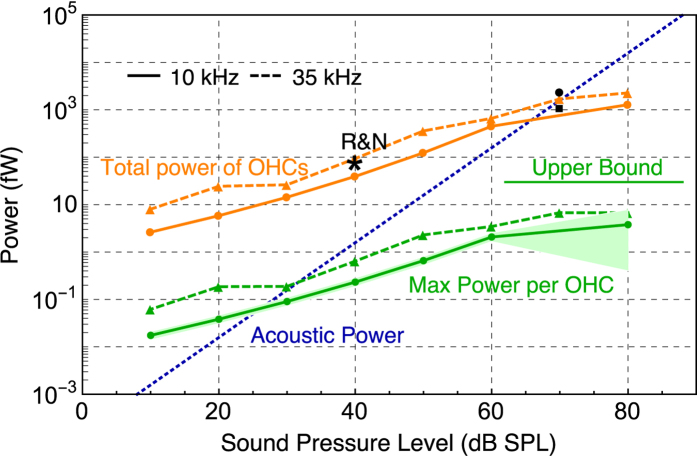
Power output versus input SPL for 10 kHz (solid orange and green lines) and 35 kHz (dashed orange and green lines) stimuli. The maximum power output per OHC (green lines) and the total power output of the OHCs (orange lines) increase with SPL and saturate above around 70 dB SPL. The total power from the OHCs is much greater than the input acoustic power (blue dashed line) for low SPLs. The present results are consistent with the estimate from Ramamoorthy and Nuttall 2012^28^ (shown as a black star) for guinea pig at 19 kHz and 40 dB SPL. The sensitivity of the total power from the OHCs to fluid viscosity is indicated by black round and square markers corresponding to twice and half the viscosity of water respectively, at 70 dB SPL for a 35 kHz stimulus. The shaded green region shows the the sensitivity of the power calculation for a 10% change in BM displacement. The upper bound OHC power output (horizontal green line) is calculated from OHC constrained force and unconstrained velocity measurements (see text).

Assuming an average center-to-center spacing of the OHCs in the longitudinal direction of 8 μm^24^ and 3 longitudinally oriented rows of OHCs, the power output of the OHCs per unit length of the cochlea (green curves in Fig. 4) can be used to estimate the power output for each OHC (*P*_OHC_) from base to apex along the length of the cochlea. To obtain the absolute power output rather than the normalized power, measured displacements of the stapes at the same frequency are used^27^. Figure 5 shows the power output per OHC along the length of the cochlea for 50 dB SPL (*α* = 0.06) and 70 dB SPL (*α* = 0.04) at 35 kHz. The peak *P*_OHC_ is estimated to be 2.3 fW and 6.8 fW at 50 dB SPL and 70 dB SPL respectively (1 fW is 10−15 W), and the location of the peak is more basal for the higher input level. From Fig. 5, it can be seen that when the SPL is low, only a small focused region of OHCs provides mechanical power along the BM. As the input SPL increases, however, there is more spread of the working OHCs, providing increased total power. In contrast to the opposite trends between the normalized power and SPL (green lines throughout Fig. 4b,d), the absolute power output increases with increasing SPL. This can be seen more clearly in Fig. 6, where the power output per OHC is plotted against the input SPL.

Figure 6 shows the total power generated by the OHCs, calculated by integrating the OHC power output per unit length over the length of the cochlea (orange lines), as well as the peak power output per OHC (green lines), together with the acoustic power input at the stapes (dashed blue line), all versus the input SPL, for both 10 kHz (solid green and orange lines) and 35 kHz (dashed green and orange lines). The calculation of the acoustic power input at the stapes is documented in Supplementary Information. The estimation of the total OHC power output in previous work^28^ for guinea pig at 40 dB SPL with a 19 kHz stimulation is also shown in Fig. 6 for comparison (black star). The peak power per OHC increases (green lines) with SPL and saturates at around 7 fW at 70 dB SPL, while the total power (orange lines) continues to increase and saturate more slowly due to the spreading of the OHCs’ power output as seen in Fig. 5. It can be seen that the total power output of the OHCs is significantly higher than the acoustic input power below about 65 dB SPL. At higher input levels, however, the total power output of the OHCs starts to saturate and becomes negligible in comparison to the increasing input acoustic power.

To investigate the sensitivity of the power calculation to fluid viscosity, the total OHC power output for two other viscosities, *μ*_water_/2 and 2*μ*_water_, are also shown in Fig. 6 (as black square and round markers respectively) at 70 dB SPL and for a 35 kHz stimulus. When the fluid viscosity is changed, the model BM amplitude changes accordingly. Consequently, the *α–*SPL mapping must be redetermined from data. The details of this calculation are given in Supplementary Information. The total power output of the OHCs for *μ*_water_/2 is 0.59 times the value for *μ*_water_, while the value for 2*μ*_water_ is 1.37 times the value for *μ*_water_. Compared to the dependency on SPL, the exact value of the viscosity within a reasonable range has little effect on the trends of the power analysis above and the conclusions of this study.

The sensitivity of the power calculation to data fitting is also investigated. At each SPL, a change in ±10% of the peak BM displacement is used as an indication of sensitivity of power calculation to the *α*–SPL mapping at 10 kHz. The power calculated using the higher and lower value of displacement is shown in Fig. 6 where the area between the higher and lower values is shaded in light green. At low SPLs, the power calculation is insensitive to 10% variation in the mapping and in the measurements. At 80 dB SPL, the acoustic power dominates the power of the OHCs, and consequently the calculation is more sensitive to changes in the measured BM displacement. The effect of *α* on the amplitude of the BM movement is smaller despite the higher power output. This makes a 10% change in the total BM movement harder to achieve except by varying *α* by a larger amount. For example, at 80 dB SPL, *α* is changed from 0.02 to 0.003 to make a −10% change in peak BM displacement.

The upper bound of the OHC power output is marked by a horizontal line in Fig. 6. The maximum power output of a piezoelectric cylinder is the constrained force times the unconstrained velocity divided by four. The measured maximum force generated by the constrained OHC is 53 pN/mV, and the unconstrained displacement is 1 nm/mV^29^. At 80–90 dB SPL, the intracellular potential fluctuation is taken as 6 mV^30^. This yields a value of 30 fW.

## Discussion

In this work, the estimation of the OHC power output is derived from the mechanical energy balance calculated using a box model of the cochlea, with anatomical representation of the BM, viscosity of the fluid in the cochlear ducts, and an approximate representation of the longitudinal cytoarchitecture of the OoC. This is, to our knowledge, the first work that separately examines the individual and collective OHC power gain, viscous power loss, and net power change. Each of these quantities provides important insights. From calculations based on the present 3D model and FF/FB mechanism, it is shown quantitatively that the OHC power output and viscous loss within the fluid both increase with SPL, while the net amplification diminishes. From the calculations, the peak of the OHC power output is slightly basal to the peak of the viscous loss. This offset, for the low SPL cases, produces a region with positive power change located basal to the BF location (the region where the blue curve is positive in Fig. 4d), which agrees with the location of negative damping in previous work^7^. In the previous literature, however, only the net power change has been studied. The OHC power gain and viscous loss are hidden within the net gain. Previous work^16^ shows that the region with negative damping diminishes with increasing SPL and vanishes in the passive case. De Boer and Nuttall commented that “with higher levels of stimulation the degree of activity of the cochlea diminishes”^7^. This is, in fact, insufficient grounds to draw conclusions on the trend of OHC power generation or viscous energy loss solely from diminishing negative damping as SPL increases. Furthermore, the common notion that the cochlear amplifier reduces damping misses the point, since greater damping is actually one of the outcomes of a working cochlear amplifier. The purpose of the cochlear amplifier is in essence to provide adequate stimulation to the IHCs by amplifying the movement of the BM at the appropriate location. Higher viscous loss is an inevitable byproduct of this mechanism.

From the current calculation, at 10 dB SPL, there is over three orders of magnitude greater power output from the OHCs than from the acoustic power input at the stapes (Fig. 6). Even though the normalized power output of the OHCs decreases as the input SPL increases (Fig. 2), the absolute OHC power output increases and saturates with increasing SPL (Fig. 6). This is consistent with the sigmoidal shape of the OHC trans-membrane voltage (or current) versus hair-bundle displacement relationship measured *ex vivo*^30^,^31^. The increasing level of stimulation induces larger hair-bundle displacement, and thus a higher level of OHC activity. However, this power output is compressive and saturates as the trans-membrane voltage (and current) plateaus in response to further increases in hair-bundle displacement. This observation is also consistent with the saturating voltage measured in the ST at high SPLs^13^.

The maximum force generated per OHC is derived from the present model to be 130 pN and 390 pN at 10 dB SPL and 60 dB SPL respectively, both at 35 kHz. This is comparable to the estimation of 200 pN at 30–40 dB SPL and 20 kHz for gerbil^13^. According to estimations based on *ex vivo* measurements^32^, the voltage fluctuation required for this force generation is on the order of 1 mV, which is also within the physiological range^32^,^33^.

The simplified box model in this study incorporates physiological parameters without any lumped mass or artificial damping. The BM stiffness and fluid viscosity are consistent with experimental measurements^18^,^34^. At first glance, one might suspect that energy loss would be strongly dependent on the fluid viscosity. However, a sensitivity analysis (Figs 6 and S1 in Supplementary Information) shows that while the viscosity affects cochlear and OHC power estimates, in comparison to the dependence on input SPL, change in fluid viscosity has little effect on the order of magnitude of the power calculated.

The WKB solution method with Fourier series, used in the present study, provides smooth solutions to the distributions of the pressure, velocity, and BM movement in the full 3D space with low computational cost. The smoothness of the solutions is advantageous for the numerical integration needed to calculate the power on a surface. To capture the concentrated distribution of pressure and velocity shown in Fig. 3 using a finite-element model would require an extremely fine mesh in the boundary layer, which would be computationally costly. The results (Fig. 3) exemplify the 3D nature of the fluid–structure interaction problem of cochlear mechanics, verified through experimental measurements^12^,^13^ and model calculations^21^,^35^. Therefore, a 3D model with fluid damping is necessary to capture key features of cochlear mechanics and power flow.

The single free parameter in the model is the OHC force-conversion factor, *α*, which is used to relate the OHC activity to the input SPL in the FF/FB model, and is determined for different input SPLs by comparison with experimental data. The incorporation of the cytoarchitecture of the OoC in the FF/FB model and the use of values of *α* less than 0.12 together provide a shift of the BF location and a gain in the BM displacement magnitude of more than 2000 times relative to the stapes displacement, which is consistent with the experimental data in Fig. 2 and other published measurements in mouse^36^. The *α* factor has been previously calculated based on measurements to be up to 0.5 under axially constrained conditions^22^,^37^.

The nonlinearity of cochlear amplification is thought to be mainly due to the nonlinear mechanics of the mechano-electro-transduction (MET) channel in the OHC hair bundles. For low-level inputs, the OHC trans-membrane voltage (or current) versus hair-bundle displacement is within the linear region of the sigmoidal-like activation curve. For high-level inputs, the OHC trans-membrane voltage and current saturate and the passive mechanics dominate. Thus, the linearization of the OHC response is a good approximation for both low and high stimuli. For intermediate inputs, however, the nonlinearity occurs within each cycle of excitation, which makes the approximation of a level-independent constant *α* less precise. Furthermore, for simplicity and to minimize the number of parameters, *α* is treated as a constant through the entire length of the cochlea at each SPL. By using a constant *α* for each SPL, the power calculations described are for a family of “linear” models with different *α* values, which together approximately characterize a nonlinear cochlea^38^. A more accurate alternative would be to have *α* depend on the local shear force on the BM, and/or to include a nonlinear electro-physiological OHC model^39^,^40^,^41^,^42^ in which the MET channel and the piezoelectric behavior are directly simulated. These more sophisticated alternatives, however, would introduce more free parameters that would need to be fitted. The simplification adopted in the present work is advantageous in avoiding parameter over-fitting, which is most suitable for the main purpose of the current work, i.e., calculating the general trend of power flow and the range of power output of the OHCs.

A basic assumption of FF/FB is that OHC motility is effective at high frequencies. The constrained OHC has been observed to produce force proportional to the intracellular voltage change up to a frequency of 80 kHz^29^. The analysis by Spector *et al*. 2003^43^ indicates that a hair-cell model including the piezoelectric properties of the wall does not show the high-frequency roll-off of intracellular voltage of simple RC circuit models. The alternate studies by Johnson *et al*. 2011^44^ and Rabbitt *et al*. 2009^45^ also indicate that OHC motility operates at sufficiently high frequencies up to 50 kHz. The measurement of Fridberger *et al*. 2004^46^ for guinea pig is compelling, showing that the electrical field near the OHC is similar to the BM displacement through 20 kHz, and that “there is no sign of a frequency-dependent decrease in amplitude”. Dong and Olson 2013^13^ simultaneously measured the electrical voltage and pressure close to the BM at a BF location of about 25 kHz in the gerbil. They show that at low levels the voltage phase leads the pressure phase basal to the BF, indicative of compressive power amplification.

Although the amplitude of the BM displacement from the model fits the data very well as shown in Fig. 2, the phase from the model rolls off faster than in the measurements near the BF location (shown in Supplementary Information Fig. S2). Similar behavior has been observed in gerbil with WKB+FF/FB, although the approach has been more successful at fitting both the amplitude and phase for chinchilla and guinea pig^19^,^20^. The disagreement in phase for gerbil and mouse is suspected to be due to the unusual thickening of the pectinate zone of the BM^47^, which has been ignored in the current study. The power from an OHC has a roughly linear dependence on the wavenumber *n* (Eq. 32–33 in the Methods). A faster phase roll-off means a larger wavenumber. At the BF location at low SPLs, the wavenumber calculated from the current model is estimated to be about 4 times larger than the actual wavenumber. Therefore, the power calculation might be overestimated by a factor of 4. This potential overestimate will lead to a small shift of the power curve in Figs 4, 5, 6. Moreover, details are omitted such as the fluid flow within the tunnel of Corti and subtectorial space^48^. Incorporating these may result in more viscous loss, such that the present work may underestimate the power generated by the OHCs.

In previous work, the somatic power of the OHCs has been estimated from an explicit piezoelectric model using biophysical parameters such as the apical and basolateral resistance and capacitance of a single OHC^41^. The total power output by a region of OHCs near the BF is reported to be about 91 fW for guinea pig at 19 kHz and 40 dB SPL^41^, which is comparable to the 39 fW for mouse at 10 kHz and 40 dB SPL calculated in the present work (Fig. 6). The method for power calculation in the present work, i.e., the use of mechanical energy balance, is an independent approach from calculations made using explicit piezoelectric models of the OHCs. Both approaches appear to provide comparable results. Moreover, in the present work a more complete analysis of OHC power output as a function of input SPL is provided.

De Boer and Nuttall^7^ employed an inverse method to calculate power flux (power on a cross section) from BM measurements using a 3D cochlear model. An important benefit of an inverse method is that it makes no assumption about the specific mechanism of the cochlear amplifier, e.g., whether it is cycle-by-cycle or if it even exists. A negative real part of the impedance was obtained, which implies that mechanical energy is pumped into the system. At low SPLs, the region of “negative damping”^7^,^14^,^15^ is basal to the BF location in their work, which is consistent with the basal location of positive net power gain in the current work. For high SPLs, the impedance comes closer to the passive impedance of the BM and becomes positive^16^. This, however, does not mean there is no energy input into the system. In fact, there is even more energy input into the system at the high level of stimulation shown in the present calculation (Fig. 6).

From the present calculation, the power on the cross section at the peak is 30 dB (1000 times) greater than the power at the stapes at 20 dB SPL for mouse, whereas with the inverse method^7^, it is only about 8 dB (6–7 times) greater at 20 dB SPL for guinea pig. The power loss in the passive case at the BF location is about 10 dB (90% loss) in de Boer and Nuttall^7^, and is only about 3 dB (50% loss) in the current study. For both passive and active cases, when compared to the current work, the previous work indicates a lower net gain and higher energy loss. This discrepancy may be due to the difficulty of the inverse procedure. An indication of the inaccuracy of the inverse method is the monotonic pressure decrease in the passive case (Fig. 1 in de Boer and Nuttall 1999^7^). However, the pressure on the BM has its maximum at the BF location even in passive cases, as is shown in our work^21^,^35^ and in measurements^49^.

In more recent work, van der Heijden and Versteegh^9^ concluded that there is no power input from the cochlea. However, their calculations of potential energy, kinetic energy, and group velocity are based on theory for conservative systems (i.e., no energy loss), leading to their conclusion that the energy loss is 30 dB (99.9% loss) at the BF for the passive case. This is not only inconsistent with a conservative system, and represents significantly more loss compared with both de Boer and Nuttall^7^ and the current work. In fact, their conclusion about the percentage of energy change is sensitive to the magnitude of the acoustic input at the stapes. The authors used the value from Ravicz *et al*.^50^, which is 20 dB larger than a more recent measurement by the same group^51^. Using the latter value leads to conclusions consistent with the present results, i.e., less energy loss for the passive and high-SPL cases and energy gain at low SPLs.

There has been significant debate as to whether the OHCs produce mechanical power in order to increase the amplitude of BM motion and improve the sensitivity and frequency selectively of hearing. The present study based on a 3D box model and FF/FB mechanism shows that the OHCs provide a significant amount of mechanical power to amplify the BM displacement. There is more viscous loss in the fluid space in an active cochlea due to larger BM and fluid motion compared to a passive post-mortem cochlea. As the SPL increases, the OHCs continue to provide larger power output. Due to the saturation of mechano-transduction, this power output, even though larger, becomes less significant in comparison to the acoustic input power at the higher levels (e.g., 70 dB SPL or more). This behavior of monotonic increase and saturation is consistent with the sigmoidal shape of the voltage–displacement relationship of the OHC. This is the first time that both the viscous power loss and OHC power gain have been shown separately and quantitatively along the length of the cochlea at different SPLs.

## Methods

The basic framework of the box model with an FF/FB mechanism and WKB solution method have been presented previously^20^,^21^. Improvements have been made in current work to the boundary conditions as in Eq. 4. Therefore, the resulting solutions in Eq. 5–11 are different from previous work^20^,^21^. The main contribution of the current work is the formulation for calculating power, which is presented at the end of the Methods. The framework for power calculation can be applied to any cochlear model, while a discussion on OHC power output specifically for models with an FF/FB mechanism is also presented at the end.

### The cochlear model and solution method

The physical cochlea is modeled as a rectangular box with rigid walls, since the coiling of the cochlea and the actual shape of its cross sections are generally considered to be of secondary importance^52^. Finite-element models that incorporate the actual shape of the cochlea provide similar results^52^. A partition divides the chamber of the box longitudinally into an upper and lower duct representing the SV and ST respectively, and the chamber is filled with fluid with the density and viscosity of water at mouse body temperature (Table 1)^18^. The anatomical parameters of the model are based on the mouse cochlea^17^,^24^, and in Fig. 1a the *x* axis denotes the longitudinal direction from base to apex, with *x* = 0 at the stapes; the *y* axis denotes the radial direction, with *y* = 0 at the medial side of the SV wall; and the *z* axis denotes the transverse direction, with *z* = 0 at the BM. The partition has two rigid sides and an orthotropic flexible plate in-between with changing width representing the BM and OoC. The dimensions and material properties of this plate yield a place-to-frequency cochlear map^53^, and the point-load stiffness of the plate is in the middle of the measured range^34^. The parameters used in the model are summarized in Table 1.

The traveling wave in the cochlea is a result of the interaction between the fluid and the cochlear partition. Due to the low Reynolds number (4.5 at 80 dB SPL), linearized fluid and plate equations are used. The resulting partial differential equations representing the fluid potentials are solved in the frequency domain using the WKB method^20^,^21^,^42^. Briefly, the fluid displacement vector **u** for incompressible viscous fluid can be expressed in terms of displacement potential


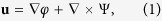


where the potentials *φ* and Ψ satisfy the following partial differential equations (PDEs)


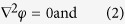



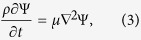


in which *ρ* and *μ* are the density and viscosity of the fluid respectively. No-slip boundary conditions (BCs) are enforced on the cochlear partition and on the ceiling of the SV. These BCs are omitted on the side walls because the dissipation happens mainly in the short-wavelength region, while the velocity on the side walls is almost zero in these regions. Thus, the BCs are


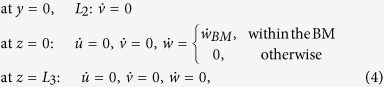


where the overhead dot denotes the time derivative, while *u*, *v*, and *w* denote the displacement in the *x*, *y*, and *z* directions respectively, and 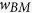 denotes the vertical displacement of the BM. The equations corresponding to the no-slip boundary condition only on the partition have been previously presented^42^.

The fluid displacement potentials that satisfy the above PDEs and BCs are


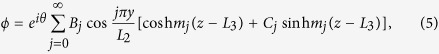



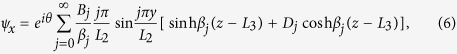



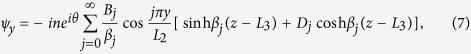


and


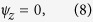


where


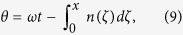



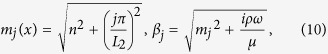



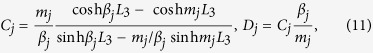


and *n*(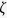) is the wavenumber at a specific location *x* = 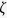, *L*_*2*_ and *L*_*3*_ are the width and height of the cochlea, *i* is the imaginary unit, and the subscript *j* denotes the mode number. In solving the PDEs (2)–(3), the derivative of *n*(*x*) in the *x* direction is neglected in the spirit of the WKB method^21^,^54^.

In the present work, the shape of the BM displacement in the *y* direction is assumed to be in the shape of a half sine wave (from 0 to π). If we let 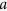 denote the beginning of the BM in the *y* direction,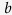 represent the width of the BM, and introduce the variable 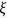 aligned in the *y* direction with 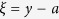, then 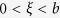 (i.e., 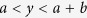) represents the region of the BM. Further, if we let 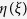 denote the half sine wave 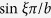, then for a specific *x* the BM displacement in the *y* direction can be written as


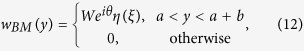


where *W* is the amplitude and *θ* is as defined in equation (9). Decomposing 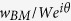 into a series of cosines in the *y* direction yields the following Fourier coefficients


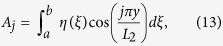


such that 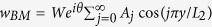. By matching the fluid displacement at *z* = 0 and the BM displacement 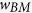, 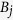 can be solved for using the orthogonality of the Fourier shape functions, such that


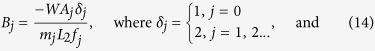



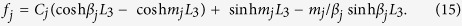


Finally, the wavenumber *n* and the amplitude *W* can be solved for considering the dynamics of the entire system using a time-averaged Lagrangian^19^,^21^, which can be written as


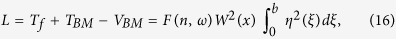


where 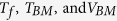 are the time-averaged kinetic energy of the fluid, kinetic energy of the BM, and potential energy of the BM as a plate, respectively. 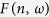 is a function of the wavenumber and input sound frequency in terms of the coefficients 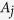, 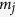, 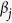, and *W*. The eikonal equation for determining the wavenumber and the equation for determining the BM amplitude are, respectively


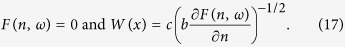


A demonstration of the solution of the traveling wave in the passive cochlea is shown in Fig. 1a.

### The active mechanism and cytoarchitecture of the organ of Corti

**Figure 7:**
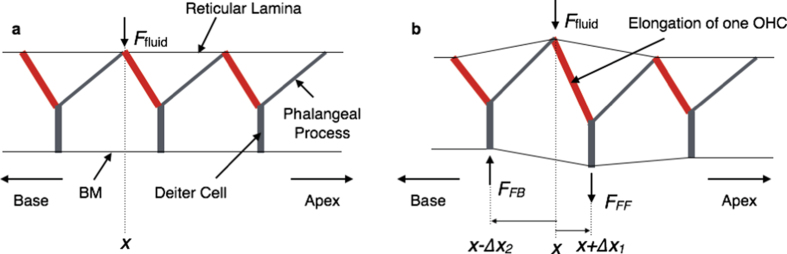
Schematic of the longitudinal view of the cytoarchitecture of the OoC without (a) and with (b) OHC elongation. **(a)** A longitudinal view of the Y-shaped structures of the inactive organ of Corti (OoC) under fluid pressure at *x*. **(b)** The effects of the feed-forward and feed-backward forces on the BM (*F*_FF_ and *F*_FB_, respectively) caused by the elongation of a single OHC. In both (**a**) and (**b**), the red bars represent the OHCs, the grey bars inclined towards the apex are the phalangeal processes, and the vertical thick grey bars are the Deiters’ cells. An OHC, phalangeal process, and Deiters’ cell combine to form a Y-shaped building block that repeats from the base to the apex of the cochlea. The elongation and shortening of the OHC in (**b**) will produce a feed-forward (FF) force at *x* + ∆*x*_1_ and an oppositely directed feed-backward (FB) force at *x* − ∆*x*_2_.

For the active mechanism, the box model above is incorporated with the feed-forward/feed-backward (FF/FB) model, which approximates the effects of the OHC forces within the cytoarchitecture of the OoC. The derivation of the FF/FB approximation has been documented^19^, and with the 3D model, the computational results found good agreement with the BM motion or neural thresholds for several animals^20^. Briefly, because of the effective “hinge” at the foot of the outer pillar cells in the OoC, pressure on the BM is fully transferred to a shear force on the stereocilia of the OHCs^55^, which modulates the opening of the ion channels of the hair bundle and consequently modulates the intracellular potential. The OHC lateral wall is effectively a piezoelectric material^22^,^37^,^43^,^56^, so the OHCs can exert force on the BM through the Deiters’ cells. Shown in Fig. 7, as a single OHC undergoes expansion, the feed-forward (FF) force at *x* + ∆*x*_1_ and the feed-backward (FB) force at *x* − ∆*x*_2_ are generated within the network of Y-shaped structures that form the basic building block of the OoC in the longitudinal direction. Each of these structures consists of an oppositely angled OHC and phalangeal process joining at the top of a Deiters’ cell^23^,^24^. The FF and FB forces acting at *x* + ∆*x*_1_ are related to the forces on the BM at *x* and *x* + ∆*x*_1_ + ∆*x*_2_ by the algebraic approximation


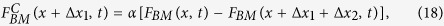


where 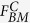 is the net force exerted by the Deiters’ cell at *x* + ∆*x*_1_ on the BM, 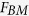
is the total force on the BM, and *α* is the OHC force-conversion factor, defined as the ratio of the force generated by the OHC over the shear force acting on the BM. Note that since the shear force on the bundle is roughly the same as the force due to pressure on the BM, *α* is also approximately the factor of the OHC axial force over the BM force as in equation (18). A detailed finite element model solution with OoC cytoarchitecture^57^,^58^ does not use this algebraic approximation, and yields similar results for the effect of the active OHCs. The FF/FB approximation is chosen in this work for its convenience in use with the WKB method. All quantities in equation (18) are expressed as the product of a slowly varying function 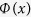 and a rapidly varying wave function as follows


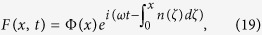


where *n* is the wavenumber in equation (5)–(11) and is also slowly varying. Therefore, for small ∆*x*_1_ and ∆*x*_2_, the following approximation can be made


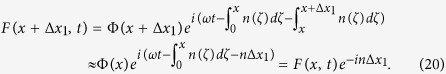


Thus, equation (20) can be written as


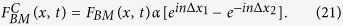


The total force acting on the BM (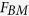) is the sum of the fluid pressure from both sides and the effective pressure from the OHCs as 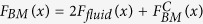

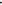
 With equation (21) and letting 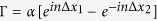, the total force on the BM is


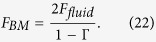


Equation (22) is only related to the dimensions of the OHCs, Deiters’ cells, and phalangeal processes, which provide the values for ∆*x*_1_ and ∆*x*_2_ in Fig. 7. The only parameter that is determined by data fitting is the OHC force-conversion factor *α*, which has been successfully determined at neural thresholds for gerbil and chinchilla. The results of the *α–*SPL mapping for mouse are shown in Fig. 2.

### Calculation of power and energy

To analyze the energy flow along the length of the cochlea, the energy balance in a thin slice of the SV fluid space at all *x* locations along the cochlea is investigated. Conservation of energy states that, in any control volume, the change in internal energy per unit time plus the change in kinetic energy per unit time is equal to the work done per unit time on the surface enclosing the volume, as follows^59^


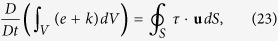


where *e* is the internal energy per unit volume, *k* is the kinetic energy per unit volume, ***τ*** is the fluid stress vector per unit area, **u** is the fluid velocity vector, and D/D*t* denotes the material derivative. All quantities in equation (23) are time-averaged over one cycle. Note that the time-averaged change in kinetic energy (*k*) at any point in space is zero. The right-hand side of equation (23) is the work done, or the power, on the closed surface enclosing the control volume. The control volume in the present work, such as the thin slice of the SV duct shown in Fig. 1, has been chosen for convenience in calculating the power output from the OoC sitting on the BM. The closed surface consists of six rectangular faces, consisting of the cross sections of the SV at position *x* and *x* + *dx* (shaded in blue in Fig. 1b), the face in contact with the cochlear partition across the SV width and with length dx (shaded in green in Fig. 1b), and three rigid walls (shaded in grey in Fig. 1b). The power on the fluid surface in contact with the BM and OoC is assumed to be the power output from the OHCs, and the power on the rigid walls is zero. The stress vector ***τ*** in equation (23) is the product of the stress tensor ***σ*** and surface normal ***n***. The components of the stress tensor are related to the fluid pressure and fluid velocity gradient by


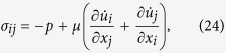


where subscripts *i* and *j* denote the *x*, *y*, or *z* direction, and *p* is the fluid pressure. The surface normal of the cross section at location *x* is defined as ***n*** = (−1, 0, 0). Therefore, the time-averaged power on the cross section, 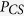, at location *x* is


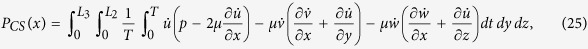


where 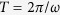 is the period of a cycle, and 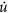, 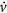, and 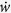 denote the velocity components in the *x*, *y*, and *z* directions respectively. To obtain the power flux on a cross section using equation (25), the fluid velocity and fluid pressure in the entire 3D fluid space are calculated from the cochlear model described above, such as is shown in Fig. 3. The power on the surface touching the BM and OoC is calculated in a similar fashion, with the integration surface consisting of a thin slice on the cochlear partition with width *L*_2_ and length d*x* (i.e., the bottom surface of the control volume in Fig. 1b), such that


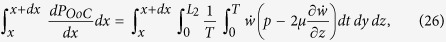


where 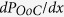 is the power input from the OoC per unit length of the cochlea. With the above specific control volume and the chosen surface normal for the cross sections, and with the fact that the time-averaged change in kinetic energy (the *k* term in equation (23)) is zero, the conservation of energy in equation (23) can be expressed as


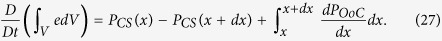


Dividing by d*x* on both sides and letting d*x* → 0, the integration over the volume becomes integration on cross-section 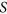, and equation (27) becomes


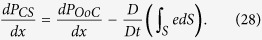


Equation (28) states that the derivative of the power on the cross section (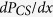) equals the power input from the OoC per unit length (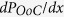) minus the energy dissipated per unit length 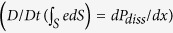 of the cochlea. 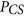 and 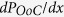 are calculated with equations (25) and (26) respectively, using numerical integration. The derivative of 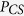 is calculated using a finite difference method. As a check of the calculation and numerical integration, the surface integration on the right-hand side of the original conservation of energy equation (23), can be rewritten as a volume integration using the divergence theorem. After simplification, the increase of the internal energy, which is also the energy dissipated due to viscosity, can be written as 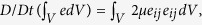 where 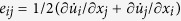 is the strain rate, and index notation is used. Dividing by *dx* and letting *dx* → 0 as before, the integrations on both sides are performed over the cross-section 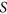. The quantity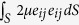 is the energy dissipation per unit length (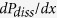). As in the previous expression in equation (28), 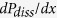 is the difference between 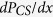 and 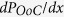. Both calculations provide identical solutions to those presented in Results.

As a final remark, the power output by the OHCs can be seen as a consequence of the FF/FB mechanism. For demonstration, at a specific *x*, the time-averaged power of the OHCs acting on the BM per unit length is one half the real part of the pressure from the OHC multiplied by the complex conjugate of the BM volume velocity per unit length


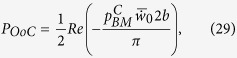


where 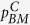 is the pressure from the OHCs in the SV, *b* is the width of the BM as usual, the top bar indicates the complex conjugate, and *w*_*0*_ denotes the displacement at the center of the BM with the positive direction pointing toward the SV (note that the area under the half sine wave 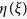 is 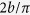). The volume stiffness *K* of the BM relates the total pressure to the displacement as


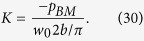


Together with equation (21), the power can be written as


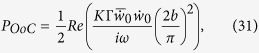


which simplifies to


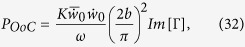


where 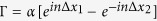 as defined before. If the small imaginary part of *n* is neglected, then


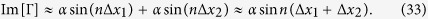


### Justifications

In this study, the differential equations and the solution method were chosen so that the essential features needed for the energy calculations could be captured, with the following simplifications and assumptions outlined below.

(1) Even though both fast and slow waves are always present in the cochlea, the fast wave is omitted in the present calculation because its effects are negligible for energy calculations. For the symmetric box model, the fast wave has symmetric pressure and displacement in the SV and ST, while the slow wave has asymmetric pressure and velocity between the two scalae. Both waves are necessary for calculation of the pressure in the ST^60^. The symmetric pressure from the fast wave produces a small net power across the cochlear partition and is therefore omitted in the energy calculations for the cochlear control volume and the stapes input^60^.

(2) The WKB solution method loses accuracy for long wavelengths. A WKB - Runge-Kutta procedure corrects this^42^. However, the effect of the correction on the present power calculations is expected to be minor^21^.

## Additional Information

**How to cite this article**: Wang, Y. *et al*. Cochlear Outer-Hair-Cell Power Generation and Viscous Fluid Loss. *Sci. Rep.* **6**, 19475; doi: 10.1038/srep19475 (2016).

## Supplementary Material

Supplementary Information

## Figures and Tables

**Table 1:** Anatomic parameters and material properties used for the cochlear model, which include the FF/FB cochlear amplification mechanism.

Description	Symbol	Value
Length of the box model^34^	*L*_1_	6.8 mm
Width of the box model^34^	*L*_2_	0.45 mm
Height of the box model^34^	*L*_3_	0.9 mm
Width of the BM^34^	*b*	63.6 μm at the base to 118 μm at the apex
Thickness of the BM^34^	*t*_bm_	14 μm at the base to 4.8 μm at the apex
∆*x*_1_ and ∆*x*_2_ in Fig. 7 for FF/FB^24^	∆*x*_1_ = ∆*x*_2_	7.7 μm at the base to 22.5 μm at the apex
Area of the stapes footplate	*A*_FP_	0.093 mm^2^
Density of the BM	*ρ*_bm_	10^3^ kg/m^3^
Density of perilymph^18^	*ρ*	10^3^ kg/m^3^
Viscosity of perilymph (~36° C)^18^	*μ*	0.0007 Pa s
