# Supplementary material for: Cochlear Outer-Hair-Cell Power Generation and Viscous Fluid Loss
Source: Sci Rep. 2016 Jan 21;6:19475. doi: 10.1038/srep19475 (PMC4726291; doi:10.1038/srep19475)
Supplement: Supplementary Information [file srep19475-s1.pdf]

Supplementary Information - Cochlear Outer-Hair-Cell Power Generation and Viscous Fluid Loss

Yanli Wang[1], Charles R. Steele[1], Sunil Puria[1,2]
1. Mechanical Engineering, Stanford University, Stanford, CA, USA
2. Otolaryngology–Head and Neck Surgery, Stanford University, Stanford, CA, USA
E-mail: puria@stanford.edu

Calculation of acoustic input power at the stapes. For the mouse model, our computed cochlear input impedance (real part) is approximately Z_{SV} = 1500 GPa·s/m^3, with the area of the stapes footplate being A_{FP} = 0.093 mm^2, as listed in Table 1. The velocity of the stapes per unit pressure at the eardrum in mouse through the middle frequencies is measured[1] to be about v_{FP} = 0.2 mm/(s·Pa). Therefore the acoustic power entering the cochlea is given by:

$$P = \frac{1}{2} Z_{SV} (A_{FP} v_{FP} p_{TM})^2 , \qquad \text{(S1)}$$

where p_{TM} is the pressure at the tympanic membrane. For 20 dB SPL at the tympanic membrane, this gives the power P = 0.01 fW, which is a point on the line for acoustic power shown in Figure 6. For comparison, the input power near the human threshold of hearing at about 8 dB SPL was estimated to be 0.015 fW at the cochlea[2].

The effects of fluid viscosity. The viscosity of the fluid in the scala vestibuli (SV) is taken as the viscosity of water at mouse body temperature (μ_{water} = 0.0007 Pa·s)[3]. To test how sensitive the present energy analysis is to the fluid viscosity, the results for viscosities a factor of two lower, $\mu_{water}/2$, and a factor of two higher, $2\mu_{water}$, are shown in Figure S1 along with the results for the nominal viscosity.

First, for a given level of basilar membrane (BM) displacement measured experimentally, the value of the force-conversion factor α needed to match the experimental data would be different for a different viscosity μ. For example, while the model with $\mu = \mu_{water}$ needs α = 0.04 to match the 70 dB SPL experimental data at 35 kHz, the model with $\mu = \mu_{water}/2$ only needs α = 0.025, and the model with $\mu = 2\mu_{water}$ needs about α = 0.06 to produce the similar level of vibration (solid lines in Fig. S1a and dashed black lines in Fig. S1b–d). The power flow for each of the three cases is shown in Figure S1b–d, which are all approximately in the same order of magnitude. The total OHC power output for these additional cases are also shown in Figure 6 as cyan and magenta stars respectively. From Figure S1, it can be seen that the power output of the OHCs per unit length (green line) and the power loss from viscosity per unit length (purple line) are both sub-linear with respect to changes in the fluid viscosity. Comparing with the dependency on the input SPL, the viscosity has little effect on the trend and order of magnitude of the power analysis above. Thus, the power analysis can be considered insensitive to the reasonable variations in fluid viscosity chosen in the present work.

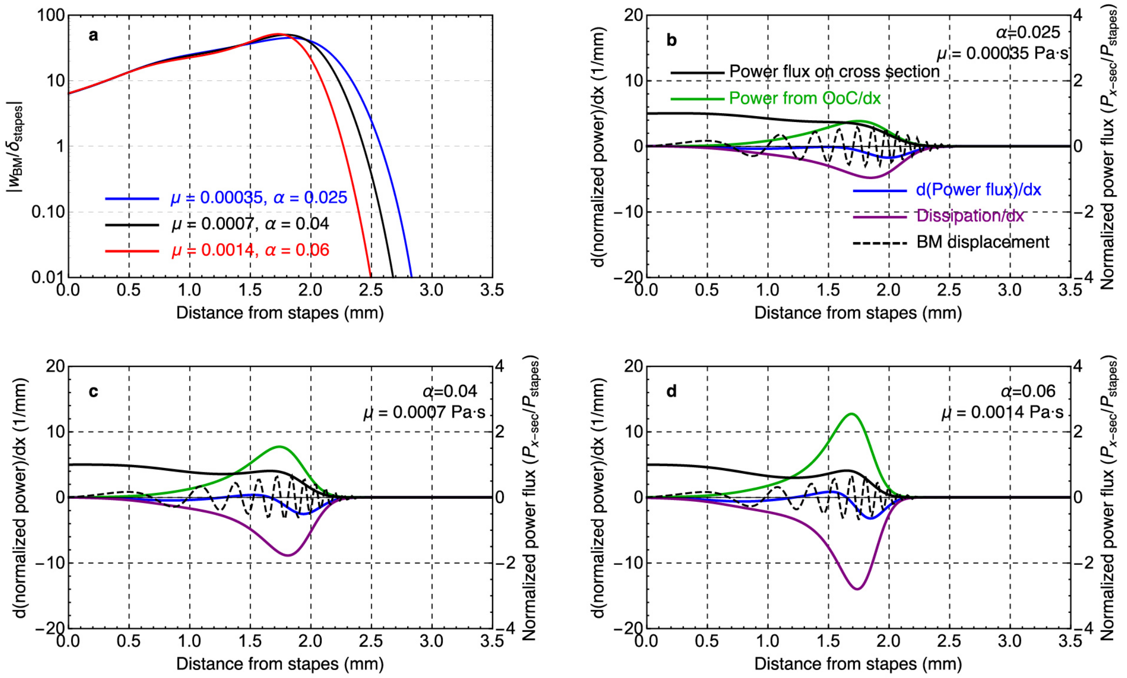

Figure S1. Comparison of power distributions along the length of the cochlea for three different viscosities at 60 dB SPL (35 kHz). Panel (**a**) shows the magnitude of the BM displacement, normalized by the magnitude of the stapes displacement, for three different fluid viscosities (μ), each with a different force-conversion factor (α). Panels (**b, c,** and **d**) contain plots of the power flow along the cochlea for each μ and α combination in (**a**). The lines represent the same quantities as in Figure 4, i.e., the power on the SV cross section (solid black lines), the derivative of the power flux along the length of the cochlea (blue lines), the power loss per unit length due to the fluid viscosity (purple lines), the power output from the OoC per unit length (green lines), and the rescaled snapshot of the BM displacement waveform (dashed black lines). Only the basal half of the cochlea is shown.

Comparison of the phase between modeling and experiments. Figure S2 shows that the phase of the BM displacement normalized by the stapes displacement from the model begins to roll off faster than the measurements near the best frequency (BF) corresponding to an 80 dB SPL input (5.5 kHz), even though the amplitude of the BM displacement from the model fits very well with the data as shown in Figure 2. Similar behavior has been observed in gerbil, although the WKB+FF/FB mechanism has been fitted successfully for both amplitude and phase for chinchilla and guinea pig[4,5]. This disagreement in phase may be due to the unusual thickening of the pectinate zone of the BM that is common to both mouse and gerbil[6,7].

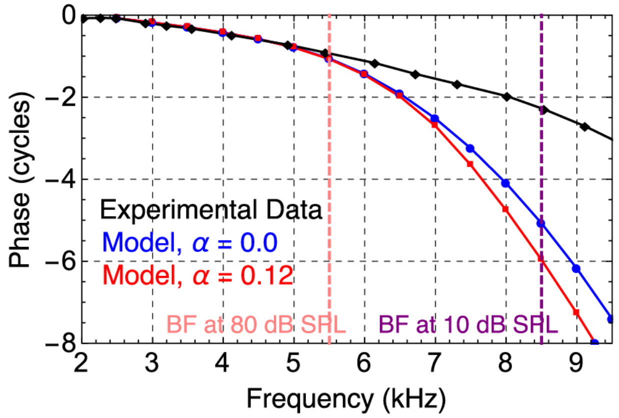

Figure S2. Comparison of phase of the BM displacement normalized by the stapes displacement between modeling and experimental results. Experimental results are shown in black markers. Results from the model are shown in blue and red for force-conversion factors α=0 and α=0.12 respectively. The pink and purple lines indicate the BF for 80 dB SPL (5.5 kHz) and 10 dB SPL (8.5 kHz) stimuli, respectively.

References

1. Dong, W., Varavva, P. & Olson, E. S. Sound transmission along the ossicular chain in common wild-type laboratory mice. *Hear. Res.* **301,** 27–34 (2013).
2. Puria, S., Peake, W. T. & Rosowski, J. J. Sound-pressure measurements in the cochlear vestibule of human-cadaver ears. *J. Acoust. Soc. Am.* **101,** 2754–2770 (1997).
3. Kate, J. H. T. & Kuiper, J. W. The Viscosity of the Pike'S Endolymph. *J. Exp. Biol.* **53,** 495–500 (1970).
4. Yoon, Y., Puria, S. & Steele, C. R. A cochlear model using the time-averaged lagrangian and the push-pull mechanism in the organ of corti. *J. Mech. Mater. Struct.* **4,** 977–986 (2009).
5. Yoon, Y.-J., Steele, C. R. & Puria, S. Feed-Forward and Feed-Backward Amplification Model from Cochlear Cytoarchitecture: An Interspecies Comparison. *Biophys. J.* **100,** 1–10 (2011).
6. Kapuria, S., Steele, C. R. & Puria, S. Mechanics of the Unusual Basilar Membrane in Gerbil. in *AIP Conference Proceedings* **1403,** 333–339 (AIP Publishing, 2011).
7. Chan, W. X. & Yoon, Y.-J. Effects of basilar membrane arch and radial tension on the travelling wave in gerbil cochlea. *Hear. Res.* doi:10.1016/j.heares.2015.06.002
